# Supplementary material for: Metabolite Analysis of Alternaria Mycotoxins by LC-MS/MS and Multiple Tools
Source: Molecules. 2023 Apr 6;28(7):3258. doi: 10.3390/molecules28073258 (PMC10096951; doi:10.3390/molecules28073258)

Supplementary Materials

Metabolite Analysis of Alternaria Mycotoxins by LC-MS/MS and Multiple Tools

Yanli You [1], Qinghua Hu [1], Nan Liu [1], Cuiju Xu [1], Sunan Lu [1], Tongcheng Xu [2,*] and Xin Mao [1,*]

[1] College of Life Science, Yantai University, Yantai 264005, Shandong, China

[2] Institute of Agro-Food Science and Technology, Shandong Academy of Agricultural Sciences, Jinan 250100, Shandong, China

* Correspondence: xtc@live.com (T.X.); maoxin@ytu.edu.cn (X.M.)

Potato Carrot Agar (PCA) Medium Preparation

40 g carrots and 40 g potatoes are separately washed, peeled, chopped, boiled in one litre water each for 5 min and filtered off. Sterilize for 15 min at 121°C. Take 250 mL potato extract and 250 mL carrot extract, 500 mL distilled water, 15 g agar and sterilize at 121°C for 15 min.

Potato Sucrose Agar (PSA) Medium Preparation

Add 200 g scrubbed and diced potatoes to 1 litre water and boil for 15 min. Let it pass through a fine sieve, add 17 g agar and 20 g sucrose and boil until dissolved. pH 5.6 ± 0.1.

Parameters for Feature Processing with MZmine 2.

Parameters were settled as follows. In Mass detection: Level 1 noise were settled at 1.0E5 and Level 2 noise were at 1.0E4. In ADAP chromatogram builder: min height intensity was settled at 1.0E5 with *m/z* tolerance at 0.02 *m/z* or 5 ppm. In Chromatogram deconvolution: m/z range for MS^2 pairing were at 0.05 Da with RT range for MS^2 at 0.10 min. In Join aligner: Weight for *m/z*: 75%; Weight for RT: 25%

Identification of *Alternaria* Mycotoxin Products.

A total of samples *Alternaria* isolates were screened for metabolite production by HPLC-HRMS/MS in both positive and negative mode. As shown in the figure below, it was found that the response value of electrospray ionization negative source was

higher than that of electrospray ionization positive source, but the metabolites peaked early in the column and retained for a short time, the positive mode could precisely avoid these problems.

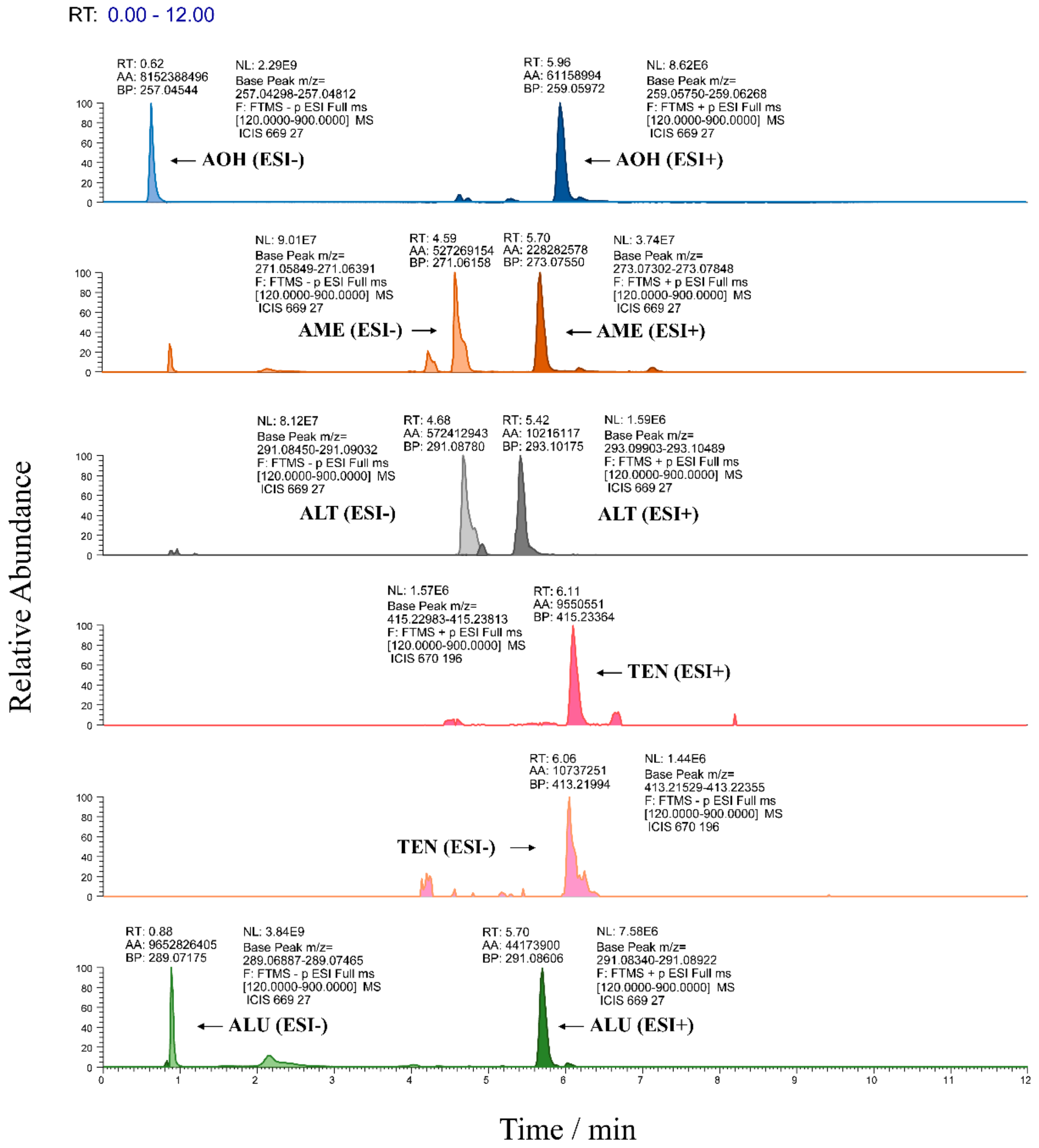

Table Captions:

Table S1. Test Fungal Strain Information.

Table S2. Gradient elution procedure of fungi culture products.

Table S3. Database of eight *Alternaria* mycotoxins

Table S4. Compounds with a neutral loss of 79.9568 Da.

Table S5. Custom mycotoxin database

Figure Captions:

Figure S1. Seven Fungi inoculated and cultured for 10 days at three different mediums (C: PCA, D: PDA, and S: PSA), (1. 11; 2. 27; 3. 72; 4. 122; 5. 196; 6. M1_2; 7. B2_1_2).

Figure S2. Chromatograms (a) and Chemical structures (b) of eight Alternaria mycotoxins standards.

Figure S3. Molecular network creation and visualization in GNPS.

Figure S4. Compound (Kynurenic acid, m/z, 190.0493, RT, 4.31) matched to GNPS-Library Spectrum CCMSLIB00005724328 (In-browser molecular network visualization).

Table S1. Test Fungal Strain Information.

Five pathogenic fungi were isolated from pear samples collected from Jinshui sluice pear garden, Jiangxia District, Wuhan City, Hubei Province, China.

Two endophytic fungi were isolated from cherry samples collected from a local supermarket in Yantai, Shandong Province, China.

No.	ID	Host	Species
1	11	Pear (Guiguan)	*Alternaria*
2	27	Pear (Eli-II)	*Alternaria*
3	72	Pear	*Alternaria*
4	122	Pear (Huangguan)	*Alternaria*
5	196	Pear	*Alternaria*
6	M1_2	Cherry (Meizao)	*Alternaria*
7	B2_1_2	Cherry (Huangmi)	*Alternaria*

Table S2. Gradient elution procedure of fungi culture products

Retention (min)	Flow (mL/min)	A%<br>0.1% Formic acid	B%<br>Acetonitrile
0.00	0.300	95.0	5.00
2.00	0.300	95.0	5.00
3.00	0.300	70.0	30.0
4.00	0.300	60.0	40.0
5.50	0.300	45.0	55.0
7.50	0.300	5.00	95.0
9.00	0.300	95.0	5.00
12.0	0.300	95.0	5.00

Table S3. Database of eight *Alternaria* mycotoxins

Compound Name	Chemical Formula	Adduct	Retention Time	Extracted Mass	Fragment1	Fragment2	Fragment3
AOH	$C_{14}H_{10}O_5$	$[M+H]^+$	5.79	259.0601	185.0597	213.0549	244.0371
AME	$C_{15}H_{12}O_5$	$[M+H]^+$	6.95	339.0169	230.0579	258.0526	212.0473
ALT	$C_{15}H_{16}O_6$	$[M+H]^+$	5.26	273.0757	257.0804	275.0909	229.0857
TEN	$C_{22}H_{30}N_4O_4$	$[M+H]^+$	5.95	353.0325	312.1703	330.1812	358.2116
ALU	$C_{15}H_{14}O_6$	$[M+H]^+$	5.59	291.0863	273.0756	255.0652	227.0702
TeA	$C_{10}H_{15}NO_3$	$[M+H]^+$	5.63	198.1124	153.0910	181.0859	125.0233
ATX-I	$C_{20}H_{16}O_6$	$[M+H]^+$	5.64	277.0706	317.0804	271.0754	335.0903
MPA	$C_{17}H_{20}O_6$	$[M+H]^+$	6.29	275.0447	207.6556	321.0339	177.0549

Table S4. Compounds with a neutral loss of 79.9568 Da

Molecular weight of Precursor Ion	Molecular Weight of Fragments Ion	Retention time	Neutral loss scan	Medium
354.2304	274.2735	7.06	79.9570	PCA
353.0324	273.0760	9.33	79.9564	PDA
399.1814	319.2246	9.28	79.9568	PDA
353.0327	273.0763	9.65	79.9564	PSA
161.9697	82.0131	3.23	79.9566	PSA
265.1074	185.1508	4.59	79.9566	PSA
184.0639	104.1068	0.95	79.9571	PSA

Table S5. Custom mycotoxin database

NO.	Accuracy Mass	Retention time	Compound Name	Molecular Formula
1	259.060	5.79	Alternariol	$C_{14}H_{10}O_5$
2	339.017	6.67	Alternariol-sulfated	$C_{14}H_{10}O_5SO_3$
3	273.076	6.95	Alternariol monomethyl ether	$C_{15}H_{12}O_5$
4	353.033	9.31	Alternariol monomethyl ether-sulfated	$C_{15}H_{12}O_5SO_3$
5	293.102	5.26	Altenuene	$C_{15}H_{16}O_6$
6	415.234	5.59	Altenusin	$C_{15}H_{14}O_6$
7	198.112	5.63	Tenuazonic acid	$C_{10}H_{15}NO_3$
8	291.086	5.95	Tentoxin	$C_{22}H_{30}N_4O_4$
9	287.056	5.64	Altertoxin-I	$C_{20}H_{16}O_6$
10	367.013	6.25	Dehydroaltenusin	$C_{15}H_{12}O_6$
11	369.027	9.65	Dehydroaltenusin-sulfated	$C_{15}H_{12}O_6SO_3$
12	275.055	6.03	Desmethyldehydroaltenusin	$C_{14}H_{10}O_6$
13	355.012	8.34	Desmethyldehydroaltenusin-dulfated	$C_{14}H_{10}O_6SO_3$
14	321.133	6.29	Mycophenolic acid	$C_{17}H_{20}O_6$
15	367.013	9.67	dehydroaltenusin	$C_{15}H_{12}O_6$

Figure S1. Seven Fungi inoculated and cultured for 10 days at three different mediums (C: PCA, D: PDA, and S: PSA), (1. 11; 2. 27; 3. 72; 4. 122; 5. 196; 6. M1_2; 7. B2_1_2)

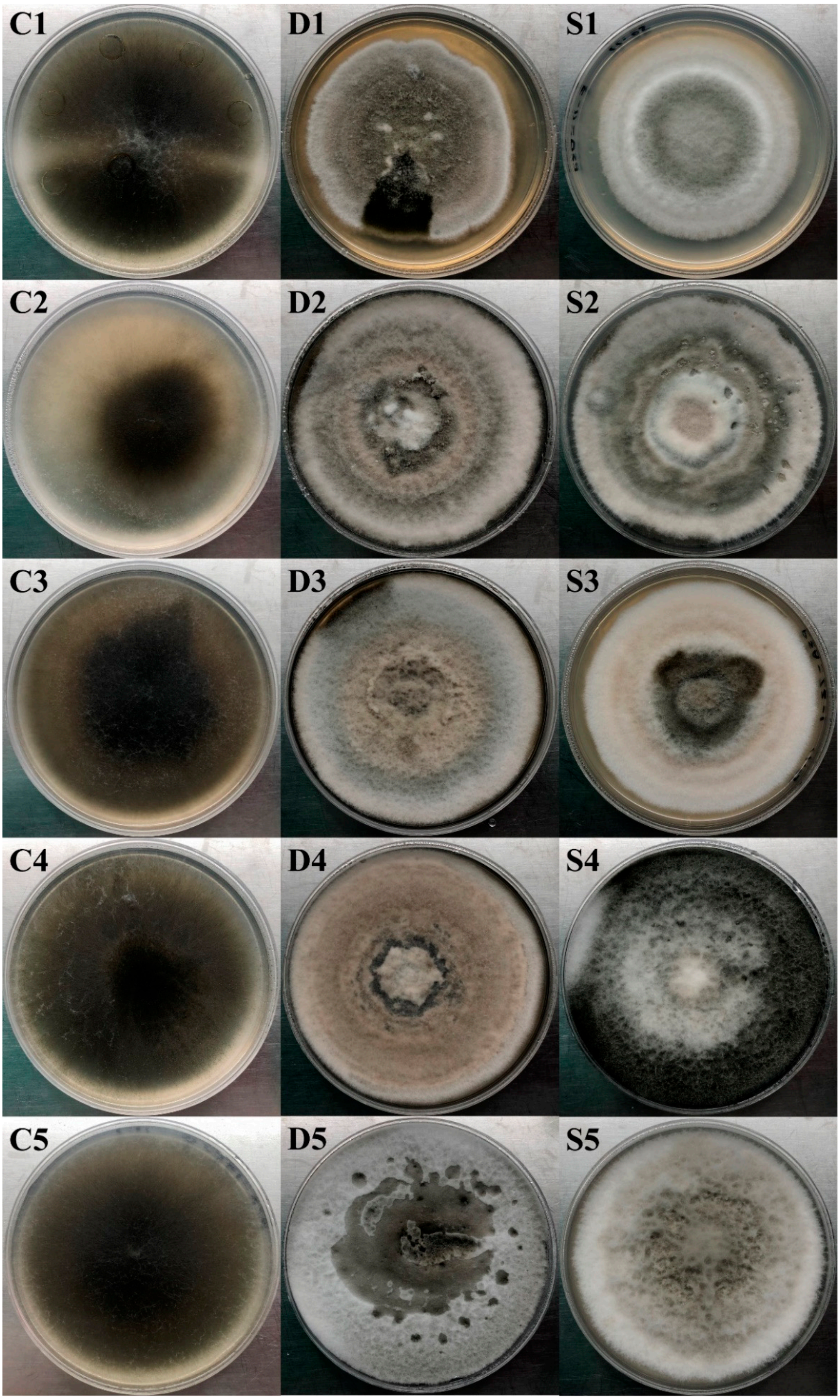

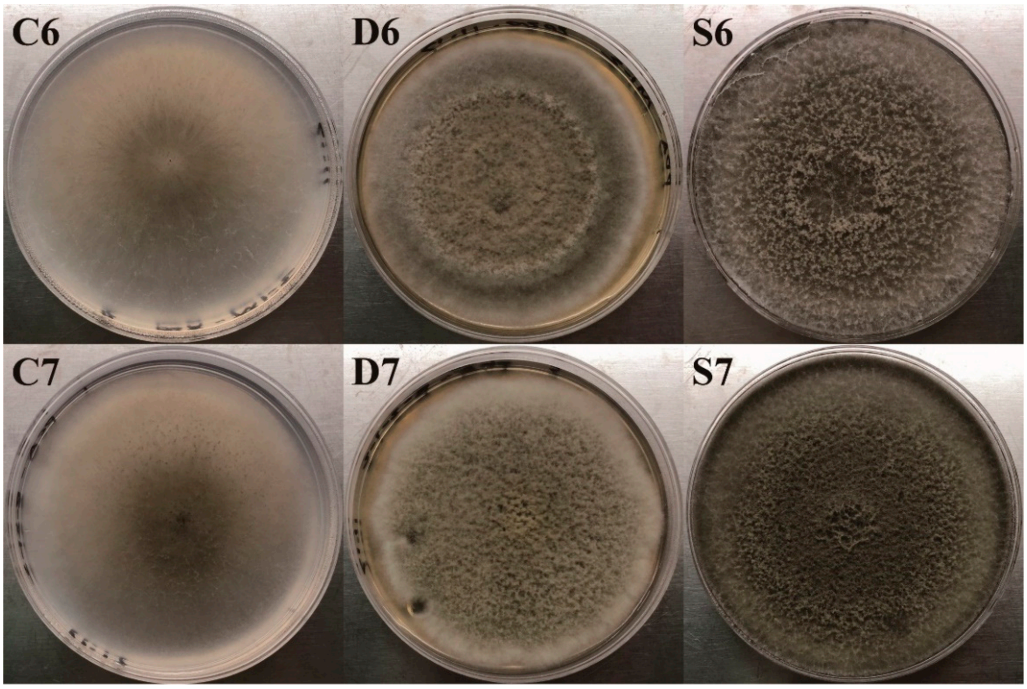
C6
D6
S6
C7
D7
S7

Figure S2. Chromatograms (a) and Chemical structures (b) of eight *Alternaria* mycotoxins standards.

a

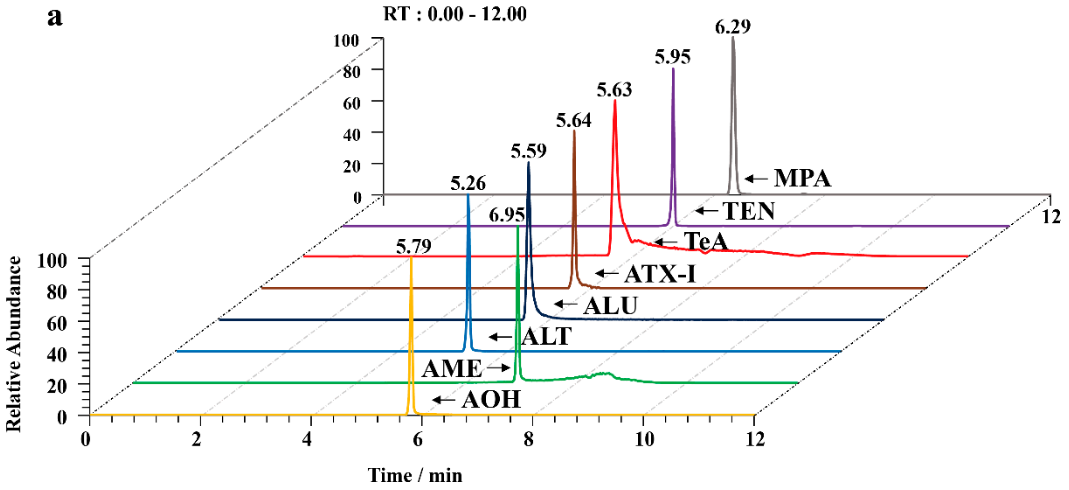

b

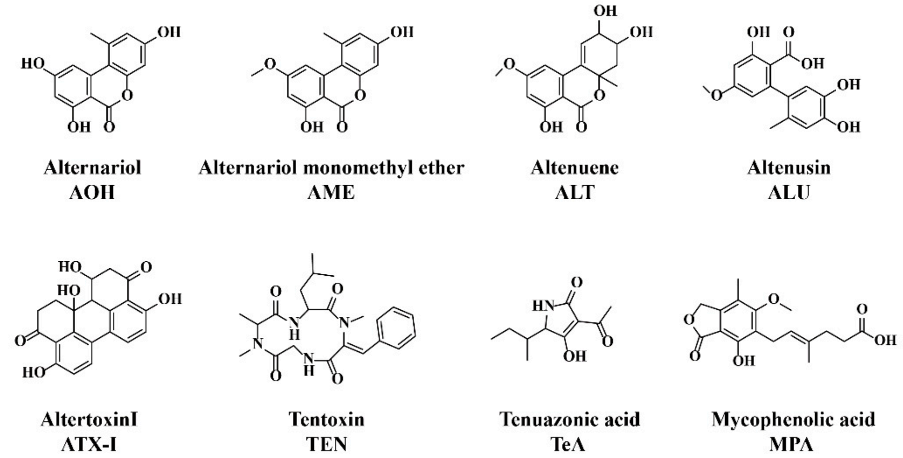

Figure S3. Molecular network creation and visualization in GNPS.

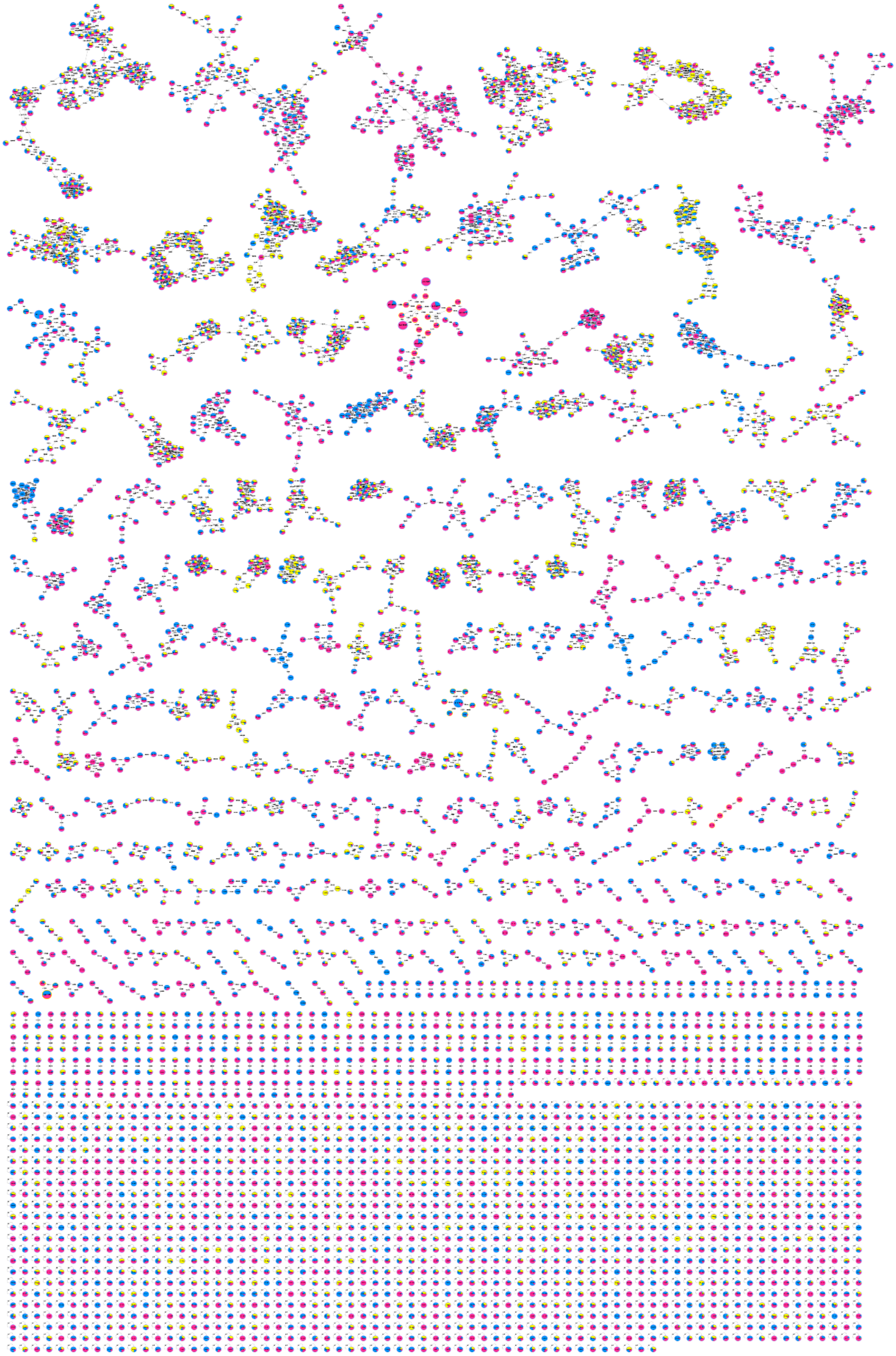

Figure S4. Compound (Kynurenic acid, *m/z*, 190.0493, RT, 4.31) matched to GNPS-Library Spectrum CCMSLIB00005724328 (In-browser molecular network visualization).

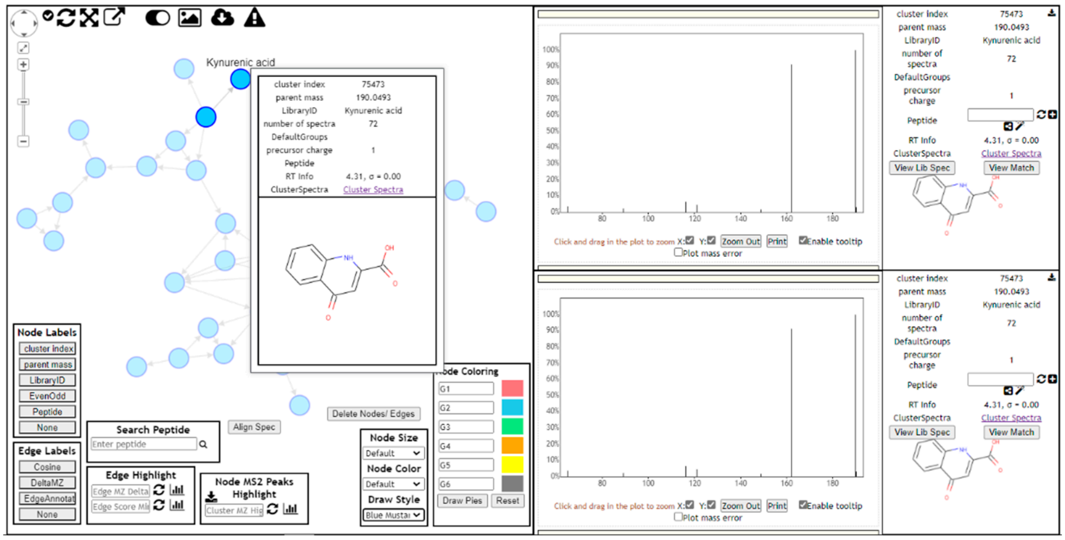

Supplement: Supplementary file 1 [file molecules-28-03258-s001.zip › molecules-2258159-supplementary.pdf]
